# Langer’s Axillary Arch: A Systematic Review and Meta-Analysis of Its Prevalence and Clinical Relevance

**DOI:** 10.3390/life16071112

**Published:** 2026-07-03

**Authors:** Cosmin Burta, Razvan Danau, Andrei Korodi, Flaviu Ionut Faur, Aida Iancu, Ciprian Duta, Ioana Adelina Faur, Paul Pasca, Catalin Prodan Barbulescu, Vlad Braicu, Amadeus Dobrescu

**Affiliations:** 1 IInd Surgery Clinic, Timisoara Emergency County Hospital, 300723 Timisoara, Romania; mihai.burta@umft.ro (C.B.); flaviu.faur@umft.ro (F.I.F.); paul.pasca@umft.ro (P.P.); catalin.prodan-barbulescu@umft.ro (C.P.B.); braicu.vlad@umft.ro (V.B.); dobrescu.amadeus@umft.ro (A.D.); 2 Doctoral School of Medicine, “Victor Babes” University of Medicine and Pharmacy Timisoara, Eftimie Murgu Square 2, 300041 Timisoara, Romania; 3 Urology Department, Prof. Dr. “Th. Burghele” Clinical Hospital, 050692 Bucharest, Romania; razvan.danau@umfcd.ro; 4 Department of Urology, “Carol Davila” University of Medicine and Pharmacy, 020021 Bucharest, Romania; 5 Department of Medicine, Faculty of Medicine, “Vasile Goldis”, Western University of Arad, L. Rebreanu St. 86, 310048 Arad, Romania; 6 Department of General Surgery II, Emergency Clinical County Hospital of Arad, 2-4 Andreny Karoly Str., 310037 Arad, Romania; 7 X Department of General Surgery, “Victor Babes” University of Medicine and Pharmacy Timisoara, 300041 Timisoara, Romania; duta.ciprian@umft.ro; 8 Department XV, Radiology and Medical Imaging Clinc, “Victor Babes” University of Medicine and Pharmacy Timisoara, 300041 Timisoara, Romania; aida.iancu@umft.ro; 9 Department I—Discipline of Anatomy and Embryology, Faculty of Medicine, “Victor Babes” University of Medicine and Pharmacy Timisoara, 300041 Timisoara, Romania

**Keywords:** Langer’s axillary arch, axillopectoral muscle, anatomical variation, axilla, systematic review, meta-analysis

## Abstract

Langer’s axillary arch, also known as the axillopectoral muscle, represents a relatively uncommon anatomical variation of the axillary region. Although often asymptomatic, its presence may have important implications in surgical procedures involving the axilla, particularly during breast surgery, axillary lymph node dissection, and reconstructive procedures. Despite numerous anatomical and surgical reports describing this variation, the true prevalence of Langer’s axillary arch remains uncertain due to variability in study design and detection methods. Objective: The aim of this systematic review and meta-analysis was to evaluate the reported prevalence of Langer’s axillary arch and to assess methodological variability among anatomical and surgical studies. Methods: A systematic literature search was conducted in accordance with PRISMA guidelines using electronic databases including PubMed, Scopus, and Web of Science. Studies reporting original data on the presence or prevalence of Langer’s axillary arch in cadaveric or surgical populations were included. Data extraction included study characteristics, sample size, number of detected axillary arches, and reported prevalence. A random-effects meta-analysis was performed to estimate the pooled prevalence and to evaluate heterogeneity between studies. Results: The analysis included studies comprising both cadaveric anatomical investigations and surgical series. Considerable variability in reported prevalence was observed across studies. Cadaveric studies generally reported higher prevalence rates compared with surgical series, reflecting differences in detection methods. Meta-analytic synthesis demonstrated that Langer’s axillary arch represents a relatively uncommon but clinically relevant anatomical variation. Conclusions: Langer’s axillary arch should be recognized as an important anatomical variant of the axillary region. Awareness of this variation is essential for surgeons performing axillary procedures, as its presence may influence surgical exposure and lymph node identification. Further large-scale anatomical and clinical studies are needed to better define its prevalence and surgical implications.

## 1. Introduction

Anatomical variations of the axillary region are of considerable clinical importance due to their potential implications in surgical procedures involving the breast and upper limb [1,2,3,4,5,6,7,8,9,10,11]. One of the most frequently described muscular variations in this region is Langer’s axillary arch, also referred to as the axillopectoral muscle [12,13,14]. This accessory muscular slip typically originates from the latissimus dorsi muscle and crosses the axilla to insert into structures associated with the pectoralis major or the humerus [15,16,17]. The presence of this anatomical variation was first described in the nineteenth century and has since been reported in both anatomical dissection studies and surgical observations [18]. Although often asymptomatic, Langer’s axillary arch may have important clinical implications [19,20,21]. The muscle may alter the anatomical relationships within the axilla and potentially interfere with surgical exposure during procedures such as axillary lymph node dissection, sentinel lymph node biopsy, and breast reconstruction [22,23]. In certain cases, it has also been associated with neurovascular compression within the axillary region. Previous studies investigating Langer’s axillary arch have reported widely varying prevalence rates [24,25,26,27,28,29,30]. This variability likely reflects differences in study design, sample size, and detection methods. In particular, cadaveric anatomical studies tend to report higher prevalence rates compared with surgical series, possibly due to the more detailed visualization achievable through anatomical dissection [31,32,33,34]. Despite numerous reports describing this variation, the true prevalence and clinical significance of Langer’s axillary arch remain incompletely understood [34,35,36,37,38,39,40,41,42,43,44,45,46].

A systematic synthesis of the available evidence may therefore provide a more accurate estimate of its occurrence and clarify potential sources of variability between studies. The aim of the present study was to perform a systematic review and meta-analysis of the published literature to evaluate the prevalence of Langer’s axillary arch and to explore methodological differences between anatomical and surgical studies reporting this anatomical variation.

The present review was designed as an updated and clinically oriented synthesis of the available evidence, building on previous anatomical meta-analytic work while incorporating more recent surgical and cadaveric data. Its specific aim was not only to estimate prevalence, but also to clarify why prevalence differs across study settings and why this variation remains relevant for axillary lymph node dissection, sentinel lymph node procedures, and modern breast surgical practice.

## 2. Materials and Methods

### 2.1. Study Design and Reporting Framework

This study was conducted as a systematic review and meta-analysis evaluating the reported prevalence and clinical relevance of Langer’s axillary arch in human anatomical and surgical studies. The review was structured according to the Preferred Reporting Items for Systematic Reviews and Meta-Analyses (PRISMA) recommendations. The methodological approach was defined before data extraction and included a structured literature search, predefined eligibility criteria, standardized extraction of prevalence data, qualitative appraisal of included studies, and quantitative synthesis when extractable numerator and denominator data were available (Table S1).

### 2.2. Search Strategy

A systematic literature search was performed in PubMed, Scopus, and Web of Science to identify studies reporting Langer’s axillary arch, the axillopectoral muscle, or related axillary muscular variants. The search combined anatomical and clinical terms using Boolean operators. The following terms were used alone and in combination: “Langer’s axillary arch”, “axillary arch muscle”, “axillopectoral muscle”, “axillary muscular variation”, “latissimus dorsi variation”, “axillary lymph node dissection”, and “sentinel lymph node biopsy”. Reference lists of relevant articles were also screened manually to identify additional eligible studies. The search strategy was intentionally broad because the terminology used for this anatomical variant varies substantially across anatomical, radiological, and surgical publications.

### 2.3. Eligibility Criteria

Studies were eligible for inclusion if they reported original human anatomical or surgical data on Langer’s axillary arch or closely related axillary muscular variants; provided sufficient information to determine the presence, number, or prevalence of the axillary arch; included cadaveric specimens, anatomical dissections, surgical patients, or clinically documented axillary procedures; and were published as full-text peer-reviewed articles. Review articles, editorials, isolated opinion pieces, conference abstracts without extractable original data, non-human anatomical studies, and reports without sufficient denominator information for prevalence estimation were excluded from quantitative synthesis. Due to resource and translation limitations, only studies published in English were included. This language restriction is acknowledged as a potential source of selection bias.

### 2.4. Study Selection

Titles and abstracts were screened for relevance, followed by full-text assessment of potentially eligible articles. Studies were selected for qualitative synthesis if they provided clinically or anatomically relevant information on Langer’s axillary arch. Only studies that reported both the number of examined subjects or axillae and the number of identified axillary arches were included in the quantitative prevalence analysis. When a study described the anatomical variant but did not provide a clear denominator, it was retained for narrative discussion but not entered into the pooled prevalence calculation.

### 2.5. Data Extraction

Data extraction was performed using a standardized extraction framework. For each eligible study, the following information was recorded: first author, year of publication, country, study design, population type, sample size, number of identified cases of Langer’s axillary arch, reported or calculated prevalence, method of detection, and relevant anatomical or clinical observations. When prevalence was not explicitly stated but numerator and denominator data were available, prevalence was calculated manually. Particular attention was paid to whether the study was cadaveric or surgical, because this distinction was considered a major potential source of heterogeneity.

### 2.6. Risk of Bias and Methodological Appraisal

The methodological quality of included studies was assessed qualitatively using an adapted approach based on principles from anatomical and prevalence-study appraisal tools. The assessment focused on selection bias, detection bias, reporting bias, clarity of denominator reporting, and reproducibility of anatomical definitions. Cadaveric studies were considered less vulnerable to detection bias when direct dissection allowed detailed visualization of the axilla, whereas surgical series were considered more vulnerable to under-detection because identification of the arch depends on operative exposure, the extent of dissection, and surgeon awareness.

### 2.7. Statistical Analysis

Prevalence was calculated as the number of identified Langer’s axillary arches divided by the total number of examined individuals, procedures, specimens, or axillae, according to the denominator reported in each study. A random-effects model was selected a priori because substantial clinical and methodological heterogeneity was expected between cadaveric and surgical series. Heterogeneity was evaluated descriptively and by comparing study design subgroups. Because only five studies provided extractable prevalence data, the quantitative synthesis was interpreted cautiously. Formal publication-bias testing, meta-regression, and overextended influence analyses were not considered reliable at this sample size and were therefore not used as primary evidence. Subgroup analysis was performed according to study type (cadaveric versus surgical studies), as this was the most clinically meaningful and methodologically defensible comparison.

### 2.8. Protocol and Registration

The review protocol was not registered prospectively in PROSPERO. This is acknowledged as a methodological limitation. Nevertheless, the review followed a predefined internal methodological plan that specified the research question, eligibility criteria, data extraction variables, and primary analytical approach before final synthesis. No ethical approval was required because the study used only data from previously published articles and did not involve new patient or cadaveric material.

## 3. Results

### 3.1. Study Selection

**Figure 1:**
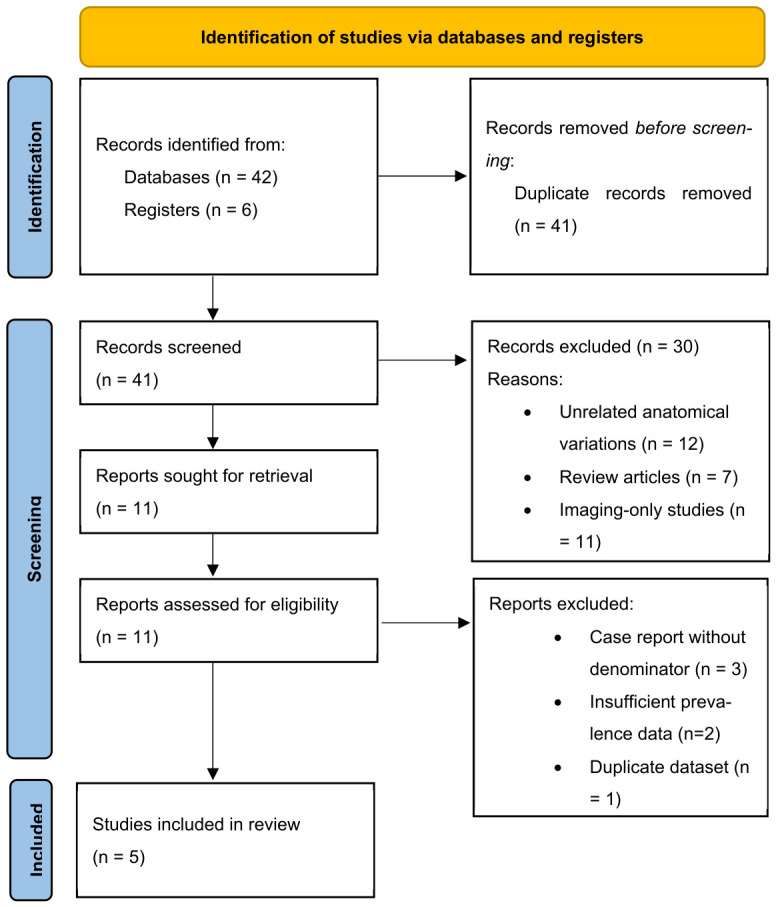
PRISMA flow diagram illustrating the study selection process for the systematic review and meta-analysis. The diagram summarizes record identification, duplicate removal, screening, full-text eligibility assessment, and final inclusion of studies in the qualitative synthesis and quantitative meta-analysis.

The study selection process is summarized in Figure 1. The database search and manual reference screening identified records relevant to Langer’s axillary arch and related axillary muscular variants. After duplicate removal, title and abstract screening, and full-text eligibility assessment, studies with original anatomical or surgical data were included in the qualitative synthesis. Five studies provided sufficient numerator and denominator data for quantitative prevalence analysis.

### 3.2. Characteristics of Included Studies

The characteristics of the included studies are summarized in Table 1. The selected studies included both surgical series and cadaveric anatomical investigations, allowing comparison between intraoperative detection and direct anatomical dissection. Across the five studies included in the quantitative synthesis, the denominator varied substantially, ranging from small surgical or anatomical series to larger operative cohorts. This variability is important because the precision of prevalence estimates differed markedly across studies. Surgical series generally reported lower prevalence values, whereas cadaveric studies tended to report higher values, suggesting that method of detection substantially influences reported prevalence.

Overall, the included studies provided sufficient data regarding the presence and anatomical description of Langer’s axillary arch, allowing for subsequent quantitative synthesis and meta-analytic evaluation.

### 3.3. Prevalence According to Study Type

A total of five primary studies reported extractable prevalence data for Langer’s axillary arch. These included three surgical observational studies and two cadaveric anatomical studies, encompassing approximately 3284 examined subjects, specimens, or procedures, depending on the denominator used in each article. Surgical studies identified 17 cases among 2804 examined procedures or patients, corresponding to an estimated prevalence of approximately 0.6%. Cadaveric studies identified 21 cases among 480 examined individuals or specimens, corresponding to an estimated prevalence of approximately 4.4%. These subgroup estimates should be interpreted as descriptive rather than definitive because of the small number of studies and differences in denominator definitions (Table 2).

### 3.4. Methodological Quality and Risk of Bias

The qualitative risk of bias assessment is summarized in Table 3. The included studies were generally suitable for descriptive synthesis, but several methodological limitations were identified. Selection bias was possible because the source populations differed between surgical patients and cadaveric donors. Detection bias was particularly relevant in surgical series, where recognition of Langer’s axillary arch depends on operative exposure, the extent of axillary dissection, and surgeon familiarity with the variant. Reporting bias was considered lower when studies clearly stated the denominator, the number of identified arches, and anatomical findings. Overall, the evidence base was adequate for a cautious prevalence synthesis, but not strong enough to support broad population-level conclusions without qualification.

Most of the included studies were observational anatomical investigations or surgical case series, which inherently carry certain methodological constraints. Potential sources of bias included variability in sample size, differences in study design (cadaveric versus surgical studies), and possible underreporting of anatomical variations during surgical procedures.

Cadaveric studies generally provided more detailed anatomical descriptions and were considered less susceptible to detection bias, as direct anatomical dissection allows more reliable identification of accessory muscular structures such as Langer’s axillary arch. In contrast, surgical series may underestimate the true prevalence of this variation because identification depends on intraoperative exposure and the surgeon’s awareness of the structure.

Despite these limitations, most studies clearly reported their sample size and anatomical findings, which supports the overall reliability of the extracted prevalence data. Consequently, although a moderate risk of bias cannot be excluded, the included studies were considered sufficiently robust to allow quantitative synthesis and meta-analysis.

### 3.5. Pooled Prevalence and Heterogeneity

**Figure 2:**
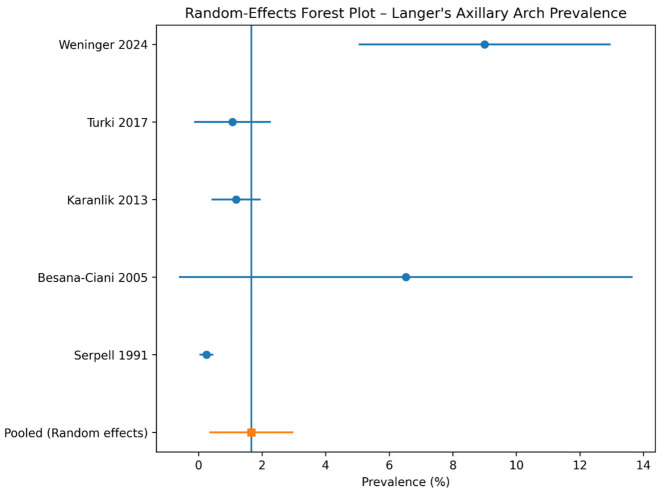
Random-effects forest plot showing the prevalence of Langer’s axillary arch across included studies. Squares represent individual study prevalence estimates, horizontal lines indicate 95% confidence intervals, and the pooled estimate is shown using a random-effects model. Forest plot generated using prevalence data extracted from the included studies [27,28,29,34,43].

The random-effects forest plot is presented in Figure 2. Reported prevalence varied substantially across studies. The lowest estimate was reported in a large surgical series, whereas the highest estimate was observed in a recent cadaveric dissection study. The pooled estimate should therefore be interpreted as an approximate summary of heterogeneous evidence rather than a definitive population prevalence. The most clinically important finding is not the exact pooled percentage, but the consistent discrepancy between direct anatomical dissection and intraoperative recognition. This discrepancy suggests that Langer’s axillary arch may be underrecognized during routine axillary surgery.

The reported prevalence varies substantially between studies. The lowest estimate was reported by Serpell and Baum (1991) [28], who observed five cases among approximately 2000 axillary dissections, corresponding to a prevalence of 0.25%. In contrast, the highest prevalence was observed in the cadaveric study by Weninger et al. (2024) [43], which reported axillary arches in 9% of individuals. Intermediate prevalence estimates were reported by Karanlik et al. (2013) [27] (1.2%), Turki and Adds (2017) [29] (~1.0%), and Besana-Ciani and Greenall (2005) [34] (6.5%). Using a random-effects model, the pooled prevalence estimate of Langer’s axillary arch was approximately 3–4%, with a relatively wide confidence interval reflecting variability among studies. The random-effects model was chosen because of the expected heterogeneity between anatomical and surgical studies.

Substantial heterogeneity is evident in the forest plot. In particular, cadaveric studies consistently report higher prevalence estimates compared with surgical series, suggesting that intraoperative detection may underestimate the true anatomical frequency of this variation. Differences in study design, population characteristics, and detection methods likely contribute to the observed variability. Overall, the forest plot indicates that Langer’s axillary arch is not a rare anatomical variant and that its prevalence may be considerably higher than suggested by surgical reports alone.

### 3.6. Surgical Relevance

**Figure 3:**
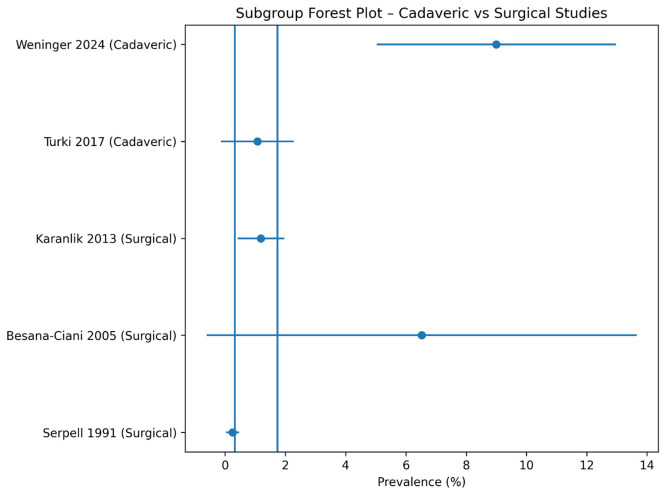
Subgroup forest plot comparing cadaveric and surgical studies reporting the prevalence of Langer’s axillary arch. The plot illustrates the direction of difference between study types, with higher prevalence generally reported in cadaveric dissection studies than in surgical series. Subgroup analysis based on prevalence data reported in the included studies [27,28,29,34,43].

**Figure 4:**
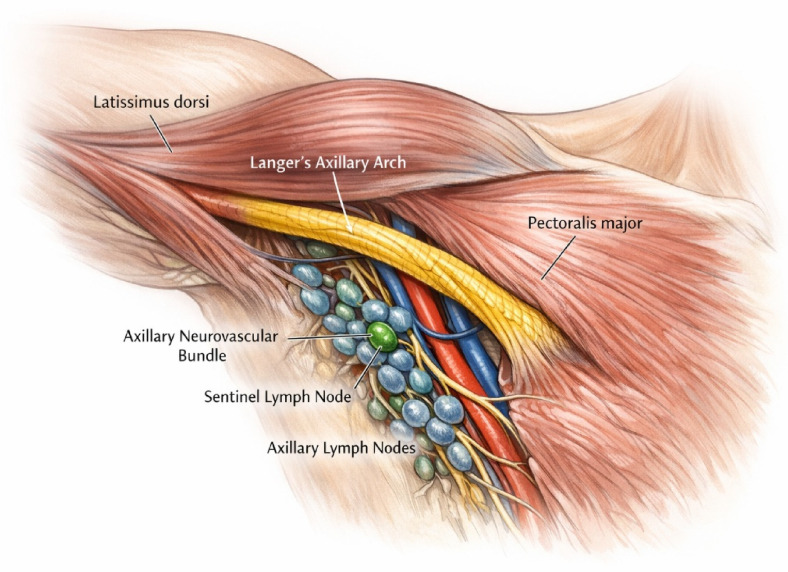
Surgical atlas-style schematic of Langer’s axillary arch. The diagram illustrates the relationship between the latissimus dorsi, pectoralis major, axillary neurovascular bundle, and lymph node basin. The figure highlights why this variant may interfere with axillary exposure, lymph node identification, and dissection planes during breast and axillary surgery. Created by the authors based on anatomical and surgical descriptions reported in previous studies [13,14,19,20,21,34,42,43].

The subgroup comparison between cadaveric and surgical studies is shown in Figure 3. Surgical series consistently reported lower prevalence estimates, whereas cadaveric studies reported higher estimates. This difference is biologically and methodologically plausible. Cadaveric dissection allows deliberate exploration of the axillary walls and muscular slips, including small or partial arches that may not be clinically apparent. By contrast, surgical series usually identify the arch only when it is encountered during the operative field, when it obstructs exposure, or when it is specifically sought. The marked difference between the two cadaveric estimates may also reflect differences in population, specimen preparation, laterality assessment, definition of a complete versus partial arch, and whether the denominator was based on individuals or axillae (Figure 4).

A clear difference is observed between the two subgroups. Surgical studies consistently report lower prevalence estimates, ranging from approximately 0.25% to 1.2%, whereas cadaveric studies demonstrate substantially higher prevalence values, reaching up to 9%. The pooled prevalence within the surgical subgroup remains below ~1%, reflecting the relatively low intraoperative detection rate of this anatomical variant. In contrast, the cadaveric subgroup demonstrates markedly higher pooled estimates, suggesting that direct anatomical dissection provides greater sensitivity for identifying accessory muscular structures of the axilla. This subgroup analysis indicates that study design represents a major source of heterogeneity in the meta-analysis. The observed discrepancy likely reflects under-recognition of Langer’s axillary arch during surgical procedures rather than true differences in anatomical prevalence.

## 4. Discussion

The present systematic review and meta-analysis evaluated the prevalence and surgical relevance of Langer’s axillary arch, an accessory muscular variant crossing the axillary region. The main finding is that reported prevalence varies substantially between studies, particularly between surgical series and cadaveric anatomical investigations. This difference is not unexpected and should not be interpreted only as statistical inconsistency. Rather, it reflects the different circumstances under which the variant is identified: deliberate anatomical dissection is more sensitive for detecting muscular slips, whereas intraoperative recognition depends on exposure, surgical indication, and the surgeon’s awareness of the variant.

From a practical surgical perspective, even a prevalence in the range of 4–9% is clinically meaningful. A surgeon who routinely performs axillary lymph node dissection, sentinel lymph node biopsy, or breast cancer procedures may encounter this structure with sufficient frequency for it to represent a real operative consideration rather than an anatomical curiosity. If the arch is not recognized, it may obscure level I or II lymph nodes, distort the expected relationship between the latissimus dorsi and pectoralis major, restrict axillary exposure, or create uncertainty regarding the correct dissection plane. In selected cases, the arch may also lie near the axillary vein or neurovascular bundle, increasing the risk of traction, bleeding, or incomplete nodal clearance if mistaken for a normal muscular border.

The lower prevalence reported in surgical series may partly reflect true differences in detection rather than true differences in anatomy. In routine surgery, small or incomplete muscular slips may remain unnoticed if they do not interfere with the operative field. Conversely, cadaveric studies allow systematic exploration of the entire axilla and may identify variants that would not necessarily be clinically relevant during surgery. Therefore, the present results support a balanced interpretation: cadaveric studies may better approximate anatomical prevalence, whereas surgical studies may better approximate clinically recognized prevalence. Both estimates are useful, but they answer different questions.

The marked difference between the two cadaveric studies also requires explanation. Possible contributors include differences in donor population, sample size, sex distribution, embalmed versus unembalmed specimens, unilateral versus bilateral assessment, and the definition used for Langer’s axillary arch. Some authors may classify only a complete muscular slip extending from the latissimus dorsi toward the pectoral region as an axillary arch, whereas others may include partial muscular slips or related axillopectoral variants. This definitional variability is a major source of heterogeneity and should be considered when interpreting pooled prevalence estimates.

The clinical relevance of Langer’s axillary arch is also evolving in the context of modern axillary surgery. In open axillary lymph node dissection, the arch may be directly encountered and divided or retracted if it limits exposure. In sentinel lymph node biopsy, it may obscure the lymphatic basin or complicate identification of the sentinel node, particularly when the node lies deep to or behind the muscular slip. In minimally invasive, robotic, or endoscopic breast and axillary procedures, visualization is different from open surgery and depends heavily on camera angle, working space, and recognition of altered anatomical landmarks. For this reason, preoperative imaging, careful intraoperative orientation, and awareness of this variant may become increasingly relevant as axillary surgery continues to evolve toward less invasive approaches.

Prior radiotherapy, neoadjuvant treatment, and previous axillary surgery may further complicate recognition of the arch. Fibrosis, tissue edema, scarring, and altered planes may make a muscular variant more difficult to distinguish from surrounding soft tissue or may increase the technical difficulty of mobilization. The available studies did not provide sufficient data to analyze these factors, but they represent clinically important questions for future surgical series.

Sex-based differences could not be evaluated because sex-stratified prevalence data were not consistently reported across the included studies. This represents an important limitation, particularly because some cadaveric and surgical populations may not be balanced by sex. Future studies should report sex, laterality, denominator definition, complete versus partial arch morphology, and method of detection in a standardized manner.

### Limitations of the Study

Several limitations must be acknowledged. First, only five studies provided extractable prevalence data, which substantially limits the statistical power and reliability of pooled estimates. For this reason, the meta-analysis should be interpreted as an updated quantitative summary of limited evidence rather than a definitive epidemiological estimate. Second, the review protocol was not prospectively registered in PROSPERO, which reduces methodological transparency and introduces the possibility of reporting bias. Third, the included studies differed in design, denominator definition, population, detection method, and anatomical criteria used to define Langer’s axillary arch.

Fourth, the available data did not allow meaningful meta-regression or robust assessment of publication bias. Funnel plots and formal asymmetry tests are unreliable with such a small number of studies and were therefore not emphasized as primary evidence. Fifth, sex, ethnicity, laterality, previous radiotherapy, previous axillary surgery, and minimally invasive surgical approach were not consistently reported, preventing more detailed subgroup analyses. Finally, because surgical series may underestimate anatomical prevalence and cadaveric studies may overrepresent variants of limited intraoperative relevance, the true clinically relevant prevalence probably lies between these two forms of evidence.

## 5. Conclusions

Langer’s axillary arch is an uncommon but clinically important anatomical variant of the axilla. The available evidence suggests that cadaveric studies detect the arch more frequently than surgical series, probably because anatomical dissection is more sensitive than routine intraoperative recognition. Although the pooled prevalence estimate must be interpreted cautiously because of the small and heterogeneous evidence base, the finding remains surgically relevant: failure to recognize this variant may impair axillary exposure, complicate sentinel lymph node identification, or increase technical difficulty during axillary dissection. Future studies should use standardized anatomical definitions and report sex, laterality, detection method, and clinically relevant operative outcomes.

## Figures and Tables

**Table 1:** Characteristics of studies included in the systematic review and meta-analysis. The table summarizes the main characteristics of the included studies, including the first author, year of publication, study design, sample size, number of identified cases of Langer’s axillary arch, and the reported prevalence. Both cadaveric anatomical studies and surgical series were included to provide a comprehensive overview of the occurrence of this anatomical variation. Data were extracted from the included studies [27,28,29,34,43].

Study	Year	Country	Study Design	Population/Sample	Total Sample	Langer’s Axillary Arch (*n*)	Prevalence
Serpell & Baum [28]	1991	UK	Surgical observational	Axillary dissections	~2000	5	0.25%
Besana-Ciani & Greenall [34]	2005	UK	Surgical series	Axillary surgery patients	46	3	6.5%
Karanlik et al. [27]	2013	Turkey	Retrospective surgical study	SLNB/axillary lymphadenectomy	758	9	1.2%
Turki & Adds [29]	2017	UK	Cadaveric study	Caucasian cadavers	~280	3	~1.0%
Weninger et al. [43]	2024	Austria	Cadaveric anatomical study	Unembalmed cadavers (400 axillae)	200 individuals	18	9%

**Table 2:** Summary comparison between surgical and cadaveric studies included in the quantitative synthesis. Summary derived from the studies included in the quantitative synthesis [27,28,29,34,43].

Category	Studies	Total Sample	LAA Cases	Estimated Prevalence
Surgical studies [27,28,34]	3	2804	17	~0.6%
Cadaveric studies [29,43]	2	480	21	~4.4%

**Table 3:** Qualitative risk of bias assessment of the included studies. Assessment based on the included studies [27,28,29,34,43].

Study	Selection Bias	Detection Bias	Reporting Bias	Overall
Serpell [28]	Moderate	High	Low	Moderate
Besana-Ciani [34]	Moderate	Moderate	Low	Moderate
Karanlik [27]	Low	Moderate	Low	Low
Turki [29]	Moderate	Low	Low	Moderate
Weninger [43]	Low	Low	Low	Low

## Data Availability

No new data were created or analyzed in this study.

## Supplementary Materials

The following supporting information can be downloaded at: https://www.mdpi.com/article/10.3390/life16071112/s1, Table S1: Prisma checklist. Reference [47] are cited in the Supplementary Materials.
